# An azo substituted quinoline‐malononitrile enzyme‐activable aggregation‐induced emission nanoprobe for hypoxia imaging

**DOI:** 10.1002/smo.20240028

**Published:** 2024-09-04

**Authors:** Zhirong Zhu, Shichang Liu, Xupeng Wu, Qianqian Yu, Yi Duan, Shanshan Hu, Wei‐Hong Zhu, Qi Wang

**Affiliations:** ^1^ School of Chemistry and Molecular Engineering Shanghai Key Laboratory of Functional Materials Chemistry Key Laboratory for Advanced Materials and Institute of Fine Chemicals Joint International Research Laboratory of Precision Chemistry and Molecular Engineering Feringa Nobel Prize Scientist Joint Research Center Frontiers Science Center for Materiobiology and Dynamic Chemistry East China University of Science and Technology Shanghai China; ^2^ School of Biomedical Engineering State Key Laboratory of Oncogenes and Related Genes Renji Hospital Shanghai Jiao Tong University Shanghai China

**Keywords:** AIE‐active, fluorescent probe, hypoxia imaging

## Abstract

The development of efficient aggregation‐induced emission (AIE) active probes is crucial for disease diagnosis, particularly for tumors and cardiovascular diseases. Current AIE‐active probes primarily focus on improving their water solubility to resist aggregation, thereby achieving an initial fluorescence‐off state. However, the complex biological environment can cause undesirable aggregation, resulting in false signals. To address this issue, we have ingeniously introduced an azo group into the AIE luminogen (AIEgen), developing a reductase‐activated AIE probe, Azo‐quinoline‐malononitrile (QM)‐PN, for imaging hypoxic environments. In this probe, the azo group promotes intramolecular motion through rapid *E*/*Z* isomerization, causing the excited state energy to dissipate via non‐radiative decay, thus turning off the initial fluorescence. In the presence of reductase, Azo‐QM‐PN is reduced and cleaved to produce the hydrophobic AIEgen NH_2_‐QM‐PN, which subsequently aggregates and generates an in situ AIE signal, thereby imaging the hypoxic environment with reductase. Encapsulation of Azo‐QM‐PN with DSPE‐PEG_2000_ results in the formation of the nanoprobe Azo‐QM‐PN NPs, which can effectively penetrate cell membranes, specifically illuminate tumor cells, monitor fluctuations in azo reductase levels, and deeply penetrate and image multicellular tumor spheroids, demonstrating potential for hypoxic tumor imaging. Additionally, the nanoprobe Azo‐QM‐PN NPs can selectively image hypoxic atherosclerotic plaque tissues, showing potential for detecting atherosclerosis. Therefore, in this study, we successfully developed an enzyme‐activated AIE probe for imaging hypoxic environments, laying the foundation for further clinical applications.

## Graphical abstract

Azo‐reductase activable AIE probe Azo‐quinoline‐malononitrile (QM)‐PN was designed to achieve visualization of hypoxia tumor. To facilitate the bio‐application of the hydrophobic probe, DSPE‐PEG_2000_ was employed to give the nanoprobe Azo‐QM‐PN NPs, which exhibited efficient inner cell delivery, desirable AIE‐active response towards hypoxia tumor cells and deep mimic tumor tissue penetrating ability, providing a promising tool for the diagnosis of tumors.

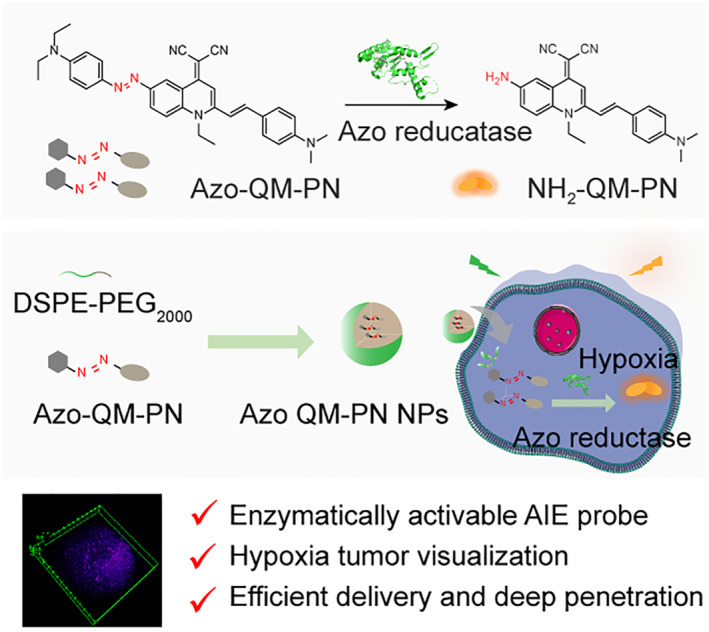

## 1 INTRODUCTION

Aggregation‐induced emission (AIE) materials have been widely used in the field of biological fluorescence detection due to their excellent optical and physical properties.[^1^, ^2^, ^3^] They have been successfully applied in active enzyme detection, organelle imaging, and fluorescence‐guided surgery.[^4^, ^5^, ^6^, ^7^] However, the current design strategy for AIE‐active probes primarily focuses on enhancing their water solubility.[^8^, ^9^] This approach facilitates the dissipation of excited‐state energy through free intramolecular motion (such as rotation and vibration), thereby achieving an initial non‐fluorescent state.[^10^, ^11^] Nevertheless, the complexity of biological environments means that these probes can undesirably aggregate in lipophilic conditions, leading to false signals.[^12^, ^13^] Therefore, it is crucial to design AIE‐active probes that do not depend on the polarity of the environment.

**FIGURE 1:**
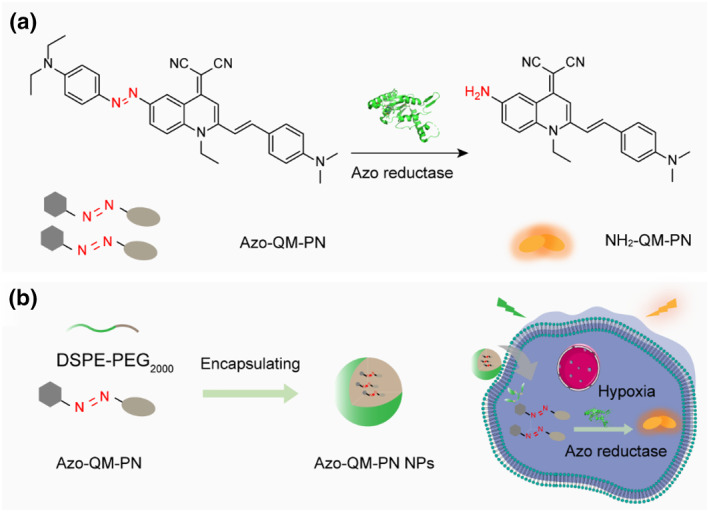
Design strategy for azo‐derived AIE‐active probes for hypoxia imaging. (a) Molecular structure of Azo‐QM‐PN and its response mechanism with reductase. (b) Encapsulation of Azo‐QM‐PN with DSPE‐PEG_2000_ to prepare AIE nanoprobe Azo‐QM‐PN NPs, enhancing cellular penetration efficiency. AIE, aggregation‐induced emission; QM, quinoline‐malononitrile.

To reduce the environmental dependence of fluorescent probes and develop probes with higher sensitivity and signal‐to‐noise ratios,^[14]^ we constructed a reductase‐activated AIE probe, Azo‐quinoline‐malononitrile (QM)‐PN (Figure 1a). This was achieved by coupling *N*,*N*‐diethylaniline with the AIE chromophore QM‐PN via an azo linkage (Figure S1).[^15^, ^16^] The azo group in this probe exhibits rapid photo‐induced *E*/*Z* isomerization, promoting intramolecular motion and dissipating excited‐state energy, thereby maintaining the probe in an initial fluorescence‐off state. In the presence of a reductive environment, the azo group (‐N=N‐) is cleaved by reductase, releasing the AIE chromophore NH_2_‐QM‐PN, which then aggregates and emits fluorescence, allowing for the visualization of the reductive environment.[^17^, ^18^] This probe's fluorescence response is less dependent on the hydrophilic or lipophilic nature of the environment, effectively enhancing detection sensitivity and signal‐to‐noise ratio.[^19^, ^20^]

Due to the rapid *E*/*Z* isomerization of the azo group, the Azo‐QM‐PN probe dissipates its excitation energy in the form of non‐radiative decay, resulting in almost no fluorescence emission in tetrahydrofuran (THF)‐water mixtures of any ratio, thus providing low background signals.[^19^, ^21^, ^22^] In contrast, its reductase cleavage product, NH_2_‐QM‐PN, tends to aggregate in aqueous solutions, producing significant aggregation‐induced fluorescence signals.[^23^, ^24^, ^25^] To enhance cellular uptake efficiency and deep tissue penetration, Azo‐QM‐PN was encapsulated with DSPE‐PEG_2000_ (Figure 1b).[^26^, ^27^, ^28^] The results showed that the encapsulated nanoparticles could be effectively taken up by tumor cells and enable imaging of hypoxic tumor cells. In tumor spheroid imaging experiments, these nanoparticles not only effectively penetrated into deep layers of the tumor spheroids but also successfully imaged the hypoxic reductive environment within the spheroids.[^29^, ^30^] Furthermore, the probe demonstrates the capability to selectively image atherosclerotic plaques by detecting the overexpression of reductase in atherosclerosis.[^31^, ^32^, ^33^] Consequently, this study successfully introduces an enzyme‐activated AIE probe tailored for imaging hypoxic environments,[^34^, ^35^, ^36^, ^37^] thereby establishing a robust platform for potential clinical applications.

## 2 RESULTS AND DISCUSSION

### 2.1 Photophysical properties

**FIGURE 2:**
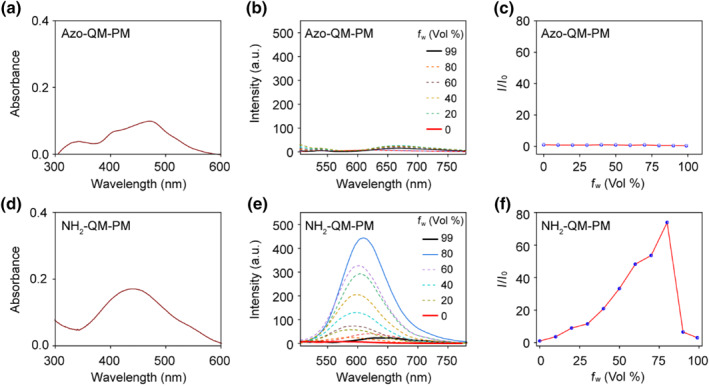
Spectral properties of NH_2_‐QM‐PN and Azo‐QM‐PN. (a, d) Absorbance of Azo‐QM‐PN and NH_2_‐QM‐PN in water, respectively. (b, e) Emission spectra of Azo‐QM‐PN and NH_2_‐QM‐PN with different water fractions (*f*_*w*_) in the mixture of the THF‐water system. (c, f) *I*/*I*_0_ plots of Azo‐QM‐PN and NH_2_‐QM‐PN, where *I* is the fluorescence intensity at 660 nm and *I*_0_ is the fluorescence intensity of Azo‐QM‐PN and NH_2_‐QM‐PN in 0% water. QM, quinoline‐malononitrile.

First, the photophysical properties of Azo‐QM‐PN and its reductase cleavage product NH_2_‐QM‐PN were evaluated.[^38^, ^39^, ^40^] The absorbance spectrum of Azo‐QM‐PN ranged from 350 to 600 nm, with a maximum absorption at 470 nm (Figure 2a). It exhibited almost no fluorescence emission in THF‐water mixtures of any ratio (Figure 2b,c). Notably, the solid‐state fluorescence emission intensity of Azo‐QM‐PN at 77 K was significantly higher than that at room temperature (Figure S2). This is likely due to the rapid *E*/*Z* isomerization of the azo group at room temperature, causing the excitation energy to dissipate through non‐radiative decay, thereby preventing fluorescence emission. Conversely, at low temperature, the restricted *E*/*Z* isomerization inhibits non‐radiative decay, restoring fluorescence. This phenomenon results in a low fluorescence background for Azo‐QM‐PN, making it advantageous for biological imaging.

The reductase cleavage product NH_2_‐QM‐PN showed absorbance in the range of 350–600 nm with a maximum absorption at 450 nm (Figure 2d). NH_2_‐QM‐PN is well‐soluble in THF and exhibits no fluorescence emission. However, as the water content (a poor solvent) increases, its fluorescence intensity rapidly increases, reaching a maximum at *f*_*w*_ = 80% (Figure 2e,f). Further increasing the water content causes the fluorescence to sharply decrease with a slight red shift (Figure 2e,f). This may be attributed to the sedimentation of AIE nano‐aggregates and the twisted intramolecular charge transfer (TICT) effect due to the increased solvent polarity. These results indicate distinct fluorescence properties of the molecules before and after the reductase response, and this turn‐on characteristic makes them suitable for further biological imaging.

### 2.2 Fluorescence responsiveness of Azo‐QM‐PN

**FIGURE 3:**
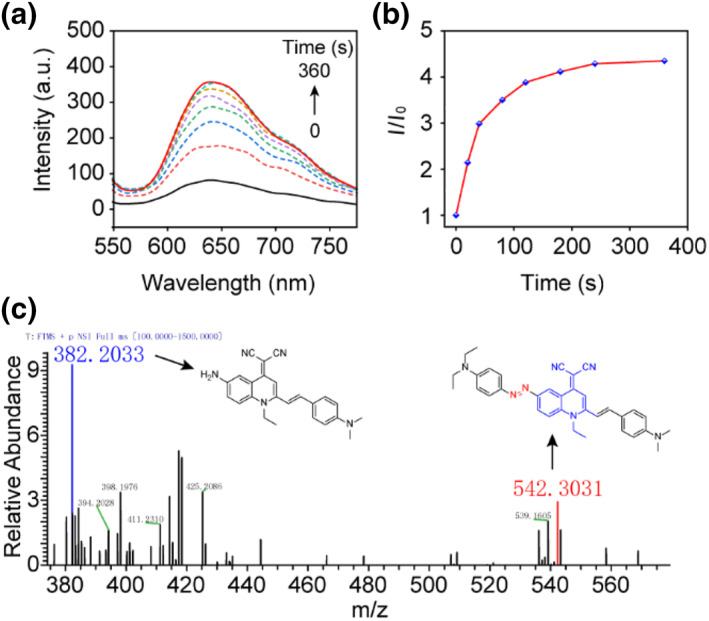
Fluorescence response of Azo‐QM‐PN to Na_2_S_2_O_4_. (a) Time‐dependent response spectra of Azo‐QM‐PN (10 μM) to sodium dithionite (50 μM). (b) *I*/*I*_0_ plot of Azo‐QM‐PN in THF/water mixtures (THF: water = 1: 4, v/v), *λ*_ex_ = 460 nm. *I*_0_ presents the initial fluorescence intensity of Azo‐QM‐PN in the mixed solution. (c) High‐resolution mass spectrometry was used to validate the degradation mechanism of Azo‐QM‐PN. QM, quinoline‐malononitrile.

Subsequently, we examined the responsiveness of the Azo‐QM‐PN probe to the reductant sodium dithionite. In the absence of the reductant, the initial fluorescence intensity was relatively low. However, upon addition of the reductant, the fluorescence intensity gradually increased over time (Figure 3a). Within the first 100 s after the addition of the reductant (Na_2_S_2_O_4_, 50 μM), a significant increase in fluorescence intensity was observed, followed by a gradual decrease in the rate of increase. By 6 min, the fluorescence intensity stabilized, reaching approximately five times the initial value (Figure 3b). Moreover, the probe also exhibits a linear relationship (Figure S3) towards the reductant with detection limit as low as 2.202 μM. High‐resolution mass spectrometry analysis detected both Azo‐QM‐PN and its reduced product NH_2_‐QM‐PN in the reaction mixture, with [M+H]^+^ peaks observed at 542.3031 and 382.2033, respectively (Figure 3c). This further confirmed that in the presence of the reductant, Azo‐QM‐PN can be converted to its reduced product NH_2_‐QM‐PN, thereby activating fluorescence.

### 2.3 Imaging hypoxic tumor cells

Hypoxia is a prominent feature of solid tumors and is closely associated with tumor recurrence and metastasis. It induces abnormal cellular metabolism and triggers oxidative stress characterized by the accumulation of free electrons and reactive oxygen species within cells. Cellular redox imbalance leads to the upregulation of reductases such as nitrate reductase and azoreductase to counteract oxidative stress. Therefore, the specific detection of reductases enables high‐fidelity imaging of hypoxic tumors, providing a viable option for the specific diagnosis of early‐stage tumors.

**FIGURE 4:**
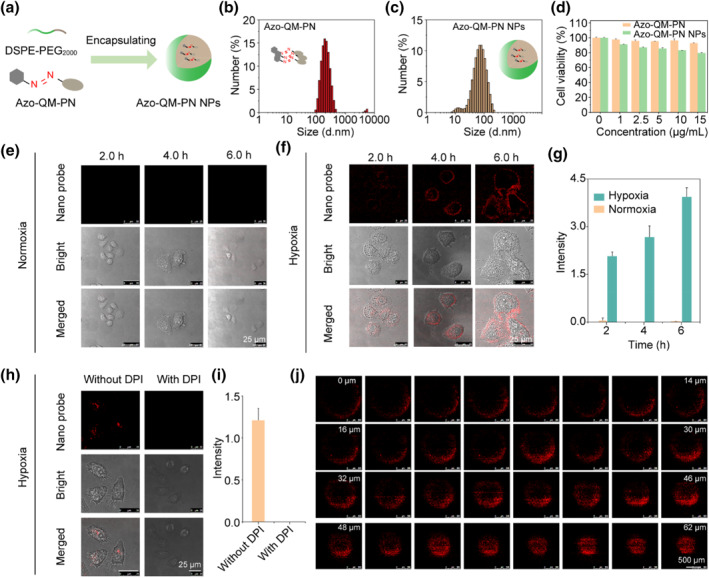
Imaging hypoxic tumor cells. (a) Preparation of Azo‐QM‐PN NPs. (b, c) Dynamic light scattering (DLS) of Azo‐QM‐PN and Azo‐QM‐PN NPs. (d) Cell viability of Azo‐QM‐PN and Azo‐QM‐PN NPs. (e, f) Imaging normoxia and hypoxia cells after incubating with Azo‐QM‐PN NPs for different times. (g) Time‐dependent fluorescence intensity of normoxia and hypoxia cells. (h, i) Hypoxic HeLa cells treated with or without diphenyl iodide chloride (DPI, an inhibitor of azoreductase) and their fluorescence intensity statistics. (j) Fluorescence images of HeLa multicellular tumor spheroids treated with Azo‐QM‐PN NPs at different depths. QM, quinoline‐malononitrile.

To enhance cellular uptake efficiency and deep tissue penetration, Azo‐QM‐PN was encapsulated with DSPE‐PEG_2000_ (Azo‐QM‐PN NPs, Figure 4a), and the size of Azo‐QM‐PN (Figure 4b) and Azo‐QM‐PN NPs (Figure 4c) were 196.9 and 53.0 nm, respectively. Azo‐QM‐PN NPs exhibits a wide range absorbance covers 400–600 nm (Figure S4), and still owns remarkable response towards Na_2_S_2_O_4_ (50 μM) (Figure S5). Cells treated with both Azo‐QM‐PN and Azo‐QM‐PN NPs exhibited good viability, indicative of good biocompatibility (Figure 4d), laying a foundation for their application in biological imaging. Herein, we then evaluated the imaging capability of Azo‐QM‐PN and Azo‐QM‐PN NPs in hypoxic HeLa cells. The results showed that the small molecule Azo‐QM‐PN (10 μM) could not illuminate tumor cells under either normoxic or hypoxic conditions. This is likely due to its high hydrophobicity, causing aggregation in the culture medium and preventing effective cell membrane penetration (Figure S6). However, the Azo‐QM‐PN nanoparticles encapsulated with the amphiphilic polymer DSPE‐PEG_2000_ exhibited good imaging capabilities in hypoxic cells (Figure 4e,f). Specifically, when Azo‐QM‐PN NPs were co‐incubated with HeLa cells under normoxic conditions, no significant fluorescence increase was observed even after 6 h of incubation (Figure 4e). In contrast, when co‐incubated with HeLa cells under hypoxic conditions, significant fluorescence was observed within 2 h, and the fluorescence intensity continued to increase with longer incubation times (Figure 4f,g). These results indicate that Azo‐QM‐PN NPs have good cell penetration ability and can specifically visualize hypoxic cells. If hypoxic tumor cells were pre‐incubated with the azo reductase inhibitor diphenyleneiodonium chloride (DPI), the fluorescence was inhibited (Figure 4h,i), indicating that the imaging of hypoxic tumor cells by Azo‐QM‐PN nanoparticles is closely related to the expression of azo reductase.

Next, we constructed 3D multicellular tumor spheroids to simulate the hypoxic microenvironment of tumor tissues and verify the imaging capability of Azo‐QM‐PN nanoparticles in hypoxic conditions. The results showed that the entire surface and interior of the tumor spheroids exhibited bright fluorescence (Figure 4j and Figure S7), indicating that Azo‐QM‐PN nanoparticles can effectively penetrate the tumor spheroids and respond to the hypoxic environment in deeper layers, demonstrating their potential for imaging deep tumor tissues.

### 2.4 Application of Azo‐QM‐PN NPs probe in imaging atherosclerotic plaques

**FIGURE 5:**
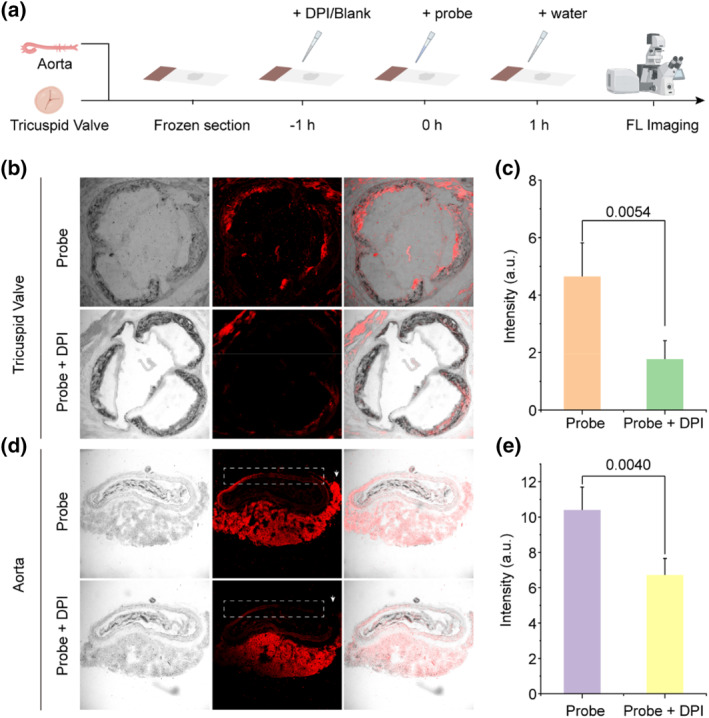
Fluorescence imaging of frozen sections of the tricuspid valve and aortic arch in atherosclerotic mice. (a) Schematic diagram of imaging. (b) and (d) Fluorescence imaging of frozen sections of the tricuspid valve and aortic arch. (c) and (e) Fluorescence intensity changes.

Atherosclerosis‐induced arterial narrowing can lead to insufficient blood and oxygen supply to tissues served by the affected arteries.[^31^, ^41^, ^42^] Similar to tumors, this hypoxic environment results in oxidative stress, which in turn upregulates reductases to counteract the stress.[^43^, ^44^] Detecting these reductases may enable specific imaging of atherosclerotic plaques.[^45^, ^46^] Therefore, we utilized Azo‐QM‐PN NPs to image atherosclerotic tissues in ApoE^−/−^ mice subjected to an 8‐week high‐fat diet. The mice were euthanized, and their tricuspid valves and aortic arches were excised, frozen‐sectioned, stained, and imaged using laser confocal microscopy (Figure 5a). Staining with the Azo‐QM‐PN NPs probe resulted in bright fluorescence signals in the tricuspid valve slices of model mice, demonstrating the probe's effectiveness in imaging atherosclerotic plaques (Figure 5b). Conversely, pre‐treatment with DPI to eliminate reductase in tissue slices led to reduced fluorescence signals in the tricuspid valve (Figure 5b,c), validating that the probe primarily images through response to reductase (Figure 5d). Similar phenomena were observed in aortic arch slices, where the Azo‐QM‐PN NPs probe effectively stained atherosclerotic plaques, and the fluorescence could be eliminated by DPI (Figure 5d and 5e). Therefore, this Azo‐QM‐PN NPs probe enables detection of atherosclerotic plaques, presenting a potential candidate for atherosclerotic plaque diagnosis.

## 3 CONCLUSION

This study focuses on the development of a novel AIE‐activated probe, breaking through the traditional design strategy to reduce the dependence of fluorescent probes on the environment, achieving high signal‐to‐noise ratio and sensitivity imaging of hypoxic microenvironments (particularly in tumors and cardiovascular diseases). By coupling *N*,*N*‐diethylaniline with the AIE chromophore QM‐PN using an azo linkage, we constructed a reductase‐activated AIE probe, Azo‐QM‐PN. The aromatic azo group in this probe exhibits rapid photo‐induced *E*/*Z* isomerization, promoting intramolecular motion and dissipating excited‐state energy, resulting in the probe being in an initial fluorescence‐off state. When a reducing environment is present, the azo group (‐N=N‐) of the probe is cleaved by reductase, releasing the AIE chromophore NH_2_‐QM‐PN, which aggregates and emits light, enabling visualization of the reducing environment. The nanoprobe Azo‐QM‐PN NPs, encapsulated with DSPE‐PEG_2000_, possesses good cell membrane penetration capability, enabling effective imaging of hypoxic tumor cells and deep imaging of multicellular tumor spheroids. Additionally, the nanoprobe demonstrated the ability to detect slices of hypoxic atherosclerotic plaque tissues. Overall, the fluorescence variation process of this probe exhibits low dependence on the hydrophilic‐lipophilic nature of the environment, effectively enhancing the detection sensitivity and signal‐to‐noise ratio, providing a promising strategy for the development of AIE‐active nanoprobes.

## CONFLICT OF INTEREST STATEMENT

The authors declare no conflicts of interest.

## ETHICS STATEMENT

No animal or human experiments were involved in this study.

## Supporting information

Supporting Information S1

## Data Availability

The data that support the findings of this study are available in the supplementary material of this article.
