# Supplementary material for: An azo substituted quinoline‐malononitrile enzyme‐activable aggregation‐induced emission nanoprobe for hypoxia imaging
Source: Smart Mol. 2024 Sep 4;3(2):e20240028. doi: 10.1002/smo.20240028 (PMC12262007; doi:10.1002/smo.20240028)
Supplement: Supplementary file 1 — Supporting Information S1 [file SMO2-3-e20240028-s001.docx]

An azo substituted Quinoline-Malononitrile (QM) enzyme-activable AIE nanoprobe for hypoxia imaging

Zhirong Zhu^1^, Shichang Liu^1^, Xupeng Wu^1^, Qianqian, Yu^1^, Yi Duan^2^, Shanshan Hu^1^, Wei-Hong Zhu^1^ and Qi Wang*^1^

^1^Shanghai Key Laboratory of Functional Materials Chemistry, Key Laboratory for Advanced Materials and Institute of Fine Chemicals, Joint International Research Laboratory of Precision Chemistry and Molecular Engineering, Feringa Nobel Prize Scientist Joint Research Center, Frontiers Science Center for Materiobiology and Dynamic Chemistry, School of Chemistry and Molecular Engineering, East China University of Science and Technology, Shanghai 200237, China.

^2^State Key Laboratory of Oncogenes and Related Genes, Renji Hospital, School of Biomedical Engineering, Shanghai Jiao Tong University, 200030, China

Contents

[**1.** **Experimental section** 2](#_Toc169710638)

[**2.** **Solid emission spectra of Azo-QM-PN measured at 77 K and room temperature** 6](#_Toc169710639)

[**3.** **Cell penetrating ability of Azo-QM-PN** 7](#_Toc169710640)

[**4.** **Reconstructed 3D tumor spheroid** 7](#_Toc169710641)

1. Experimental section

1.1 Materials and instruments

All solvents and chemicals, unless special stated, were purchased commercially in analytical grade and used without further purification. ^1^H and ^13^C NMR spectra in deuterium generation reagent were obtained with a Bruker AvanceIII 400 MHz NMR spectrometer using TMS as an internal standard. High resolution mass spectrometry (HRMS) spectra were measured with a Waters LCT Premier XE spectrometer. UV-Vis absorption and fluorescence spectra were recorded on an Agilent Cary 60 spectrophotometer and Varian Cary Eclipse fluorescence spectrophotometer (10 × 10 mm quartz cuvette), respectively. Confocal fluorescence images were performed on confocal laser scanning microscope (CLSM, Leica confocal microscope TCS SPS CFSMP).

1.2 Synthetic routes

**Figure. S1** Synthetic routes and chemical structure of AZO-QM-1.

1.3 Synthesis of Boc-QLI

2-methyl-6-BOc-aminoquinoline (4 g, 15.50 mmol) and ethane iodide (7.25 g, 46.5 mmol) were dispersed in acetonitrile (60 mL). Under nitrogen protection, the reaction was heated at 90 ℃ for 12 h. The acetonitrile and unreacted ethane iodide were removed by spin distillation, and 5.41 g brown solid was obtained by column chromatography (DCM: MeOH = 50:1), with a yield of 84.2%.^1^H NMR (400 MHz, DMSO-*d*_6_, ppm): *δ* 10.20 (s,1H, amide-H), 9.01-8.99(d, 2H, *J* = 8.8 Hz, Ph-H), 8.55-8.53(d, 2H, *J* = 11.2 Hz, Ph-H), 8.08-8.00(dd, 2H, *J* = 11.2 Hz, Ph-H), 4.93-4.92(d, 2H, *J* = 6.8 Hz, Ph-H), 3.04(s, 3H, -C***H***_3_), 1.53 (s, 9H, Boc-H), 1.12-1.08(t, 3H, *J* = 7.2 Hz, N-CH_2_C***H***_3_).

1.4 Synthesis of Boc-QM

6-((tert-butoxycarbonyl)amino)-1-ethyl-2-methylquinolin-1-ium (5 g, 12.07 mmol) and malononitrile (2.23 g, 24.14 mmol) were dispersed in ethanol (60 mL). Under ice bath conditions, sodium ethanol (0.82 g, 12.07 mmol) was slowly dripped into 80 ml of ethanol. After the reaction was completed, 2.21 g yellow solid was obtained by extraction and filtration, recrystallization with methanol, and the yield was 52.2%. ^1^H NMR (400 MHz, DMSO-*d*_6_, ppm): *δ* 9.83 (s, 1H, amide-H), 8.92 (s, 1H, Ph-H), 8.01-7.99 (d, 1H, *J* = 9.2 Hz, Ph-H), 7.92-7.90 (d, 1H, *J* = 9.2 Hz, Ph-H), 6.77 (s, 1H, Ph-H), 4.45-4.41 (t, 2H, *J* = 7.6 Hz, Ph-H), 2.64 (s, 3H, -C***H***_3_), 1.50 (s, 9H, Boc-H), 1.35-1.31 (t, 3H, *J* = 6.4 Hz, N-CH_2_C***H***_3_). Mass spectrometry (ESI positive ion mode for [M+H]^+^): Calcd. for C_20_H_23_N_4_O_2_: 351.1816; found: 351.1815.

1.6 Synthesis of Boc-QM-PN

2-methyl-6-BOc-aminoquinoline nitrile (1.5 g, 4.29 mmol) and *p*-dimethylaminobenzaldehyde (4.5g, 12.86 mmol) were dissolved in acetonitrile (30 mL) and 0.5 ml piperidine was added. Under nitrogen protection, reflux reaction was heated at 90 ℃ for 8 h. After the reaction was completed, the black solid was 0.921 g and the yield was 44.6%. ^1^H NMR (400 MHz, DMSO-*d*_6_, ppm): *δ* 9.86 (s, 1H, amide-H), 8.92 (d, 1H, Ph-H), 8.03-7.90 (dd, 2H, *J* = 9.2 Hz, Ph-H), 7.66-7.64 (d, 2H, *J* = 8.8 Hz, Ph-H), 7.35-7.18 (dd, 2H, *J* = 15.2 Hz, alkene-H), 7.00 (s, 1H, Ph-H), 6.76-6.74 (d, 2H, *J* = 8.8 Hz, Ph-H), 4.55-4.53 (d, 2H, *J* = 7.2 Hz, N-C***H***_2_CH_3_), 3.00 (s, 6H, -C***H***_3_), 1.50 (s, 9H, Boc-H), 1.41-1.37 (t, 3H, *J* = 7.2 Hz, N-CH_2_C***H***_3_). Mass spectrometry (ESI positive ion mode for [M+Na]^+^): Calcd. for C_29_H_31_N_5_O_2_Na: 504.2375; found: 504.2386.

1.7 Synthesis of NH_2_-QM-PN

Boc-QM-PN (500 mg, 1.039 mmol) was dispersed in methylene chloride (10 mL). Under the condition of ice bath and nitrogen environment, slowly add 4 mol/L HCl to 10 mL of ultra-dry solvent dissolved in 1, 4-dioxane. Add and stir at room temperature overnight. Orange solid 372 mg was obtained with a yield of 93.92%. ^1^H NMR (400 MHz, DMSO-*d*_6_, ppm): *δ* 8.33 (s, 1H, Ph-H), 8.04 (s, 1H, Ph-H), 7.74-7.71 (d, 2H, *J* = 7.2 Hz, Ph-H), 7.61 (s, 1H, Ph-H), 7.37 (s, 1H, Ph-H), 7.35-7.31 (d, 2H, *J* = 16.0 Hz, alkene-H), 7.00 (s, 2H, Ph-H), 5.77 (s, 2H, -N***H***_2_), 4.56-4.54 (d, 2H, *J* = 6.0 Hz, N-C***H***_2_CH_3_), 3.03 (s, 6H, -C***H***_3_), 1.39 (s, 3H, N-CH_2_C***H***_3_). Mass spectrometry (ESI positive ion mode for [M+H]^+^): Calcd. for C_24_H_24_N_5_: 382.2032; found: 382.2035.

1.8 Synthesis of Azo-QM-PN

NH_2_-QM-PN (372 mg, 0.98 mmol) was dispersed in a mixture of DCM (16 mL) and CH_3_CN (4 mL), followed by the addition of 0.5 mL trifluoroacetic acid. Stir the reaction mixture under an ice bath and nitrogen atmosphere for 15 minutes. Then introduce sodium nitrite (136 mg, 1.95 mmol) and continue stirring for an additional 5 minutes. Finally, add twice the molar equivalent of dimethylaminobenzene and stir for 2 hours. Upon completion of the reaction, isolate the solid by filtration and recrystallize to obtain red solid (273 mg, 51%).

1.6 Cell line

The HeLa were purchased purchased from Fuheng Biology (Shanghai, China). The cell was propagated in petri dish cultured at 37 ℃ under a humidified 5% CO_2_ atmosphere in DMEM medium (GIBCO/Invitrogen, Camarillo, CA, USA), which were supplemented with 10% fetal bovine serum (FBS, Biological Industry, Kibbutz Beit Haemek, Israel) and 1% penicillin-streptomycin (10,000 U mL^-1^ penicillin and 10 mg mL^-1^ streptomycin, Solarbio life science, Beijing, China).

**1.7 *In vitro* cytotoxicity assay**

The cell cytotoxicity of Azo-QM-PN and Azo-QM-PN NPs in HeLa cells was evaluated by MTT (3-(4,5-dimethylthiazol-2-yl)-2,5-diphenyltetrazolium bromide) assay. Briefly, cells were seeded into 96-well plates at a density of 1 × 10^4^ cells/well, and were cultured at 37ºC under a humidified 5% CO_2_ atmosphere for 12 h. Then, the cells were exposed to the various concentrations (0, 1. 2.5, 5, 10, 15 µg/mL) of Azo-QM-PN and Azo-QM-PN NPs, and for negative control group, 100 µL of culture medium were added. After incubation at 37ºC under a humidified 5% CO_2_ atmosphere for 24 h, MTT solution (5 mg/mL in PBS, 10 µL) was added to the media and incubated for another 4 h, and the absorbance at 490 nm was measured with a Multimode Plate Reader (BioTek, USA).

1.8 Cells imaging

HeLa cells were seeded onto confocal petri dishes in culture medium (1.0 mL) and allowed to adhere for 12 h before imaging. Probe zo-QM-PN and Azo-QM-PN NPs at a final concentration of 10^-5^ M (containing 2% DMSO) were added into culture medium and incubated for different time at 37℃ under a humidified 5% CO_2_ atmosphere. Cells imaging was captured by using a confocal laser scanning microscope (CLSM, Leica confocal microscope TCS SPS CFSMP) with a 60 × oil immersion objective lens. The fluorescence signals of cells incubated with probes were collected at 550-625 nm under excitation wavelength at 460 nm.

- 1. **Statistical analysis**

All statistical analyses were performed using Origin. Data were reported as the mean ± SD. The experimental data were statistically analyzed by using T-test.

- 1. **Preparation of nanoparticles**

2.0 mg DSPE-PEG_2000_ and 1.0 mg of Azo-QM-PN were dissolved in 1 mL THF. Transfer 200 μL of the resulting mixture into a separate container containing 9 mL of ultrapure water. Utilizing a cell disrupter to vigorously sonicate the mixture for a duration of 1 minute, repeating this process a total of 5 times. Once the repeated sonication is completed, continue to vigorously sonicate the mixture for an additional period of 3 minutes. THF in the mixture was evaporate overnight, yielding the solution of Azo-QM-PN NPs.

- 1. **Cell culture in hypoxia environment**

The Hela cells were seeded onto a confocal dish (NEST) and cultured overnight until they achieved full adherence. Subsequently, Azo-QM-PN NPs were introduced for co-culture. Following this, the confocal dish was placed in a 2.5 L independent culture bag and a complete hypoxia gas pack was added to create hypoxia environment, which lasted for 1 hour. After incubation times of 2 hours, 4 hours, and 6 hours respectively, the cells were washed with PBS three times and supplemented with 1 mL of culture medium. Finally, images were acquired using confocal microscopy (Leica TCS SP8, 63 × oil lens).

1. **Solid emission spectra of Azo-QM-PN measured at 77 K and room temperature**


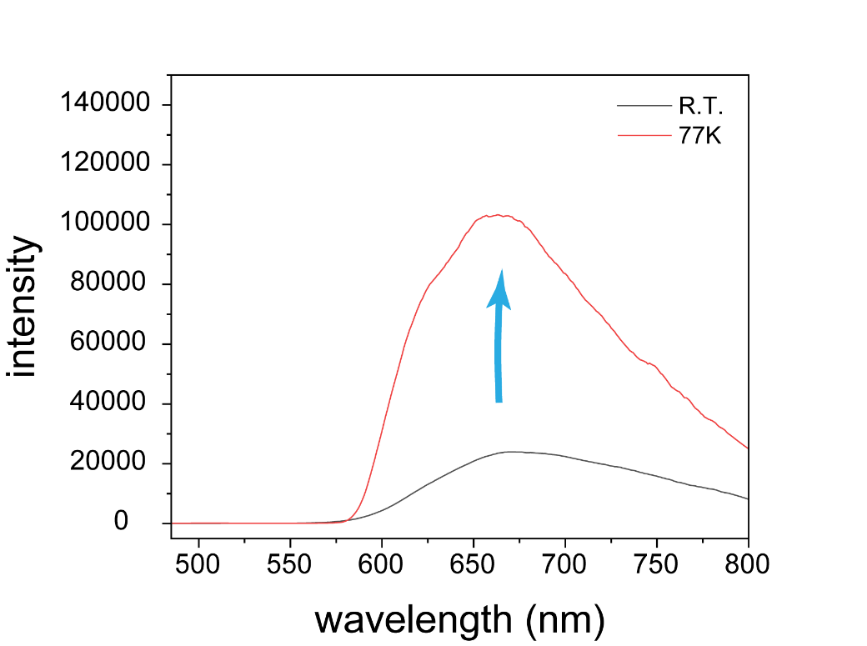


**Figure. S2** Solid emission spectra of Azo-QM-PN measured at 77 K and RT, λex = 365 nm. The low temperature can suppress the intramolecular motion, resulting an obvious emission, while the fast E-Z isomerization of Azo in room temperature will lead to non or weak emission.

1. Concentration dependent response of Azo-QM-PN towards Na_2_S_2_O_4_


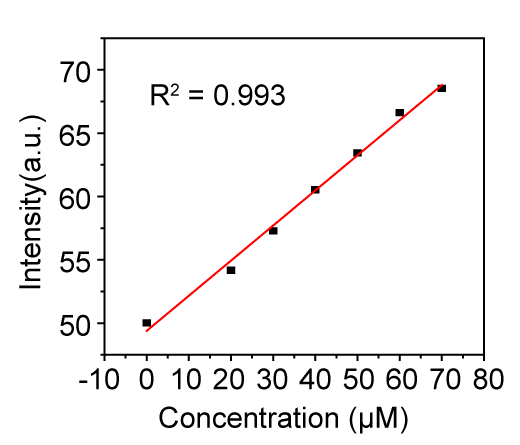


**Figure. S3** Response of Azo-QM-PN towards Na_2_S_2_O_4_ with different concentration.

1. Absorbance of Azo-QM-PN NPs


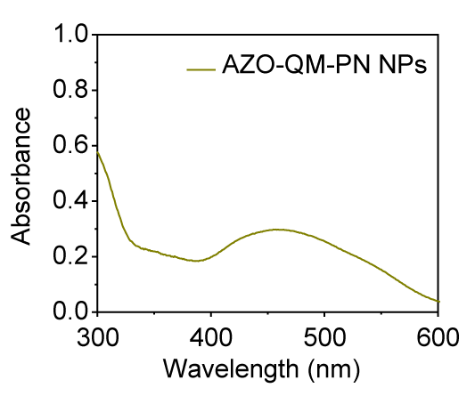


**Figure. S4** Absorbance of Azo-QM-PN.

1. Responsiveness of Azo-QM-PN NPs to Na_2_S_2_O_4_


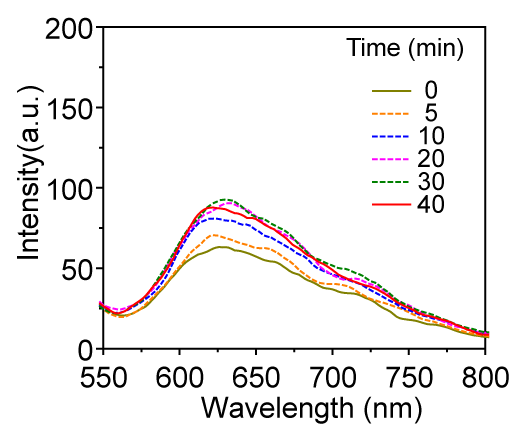


**Figure. S5** Time-dependent response spectra of Azo-QM-PN to sodium dithionite (50 μM).

1. Cell penetrating ability of Azo-QM-PN


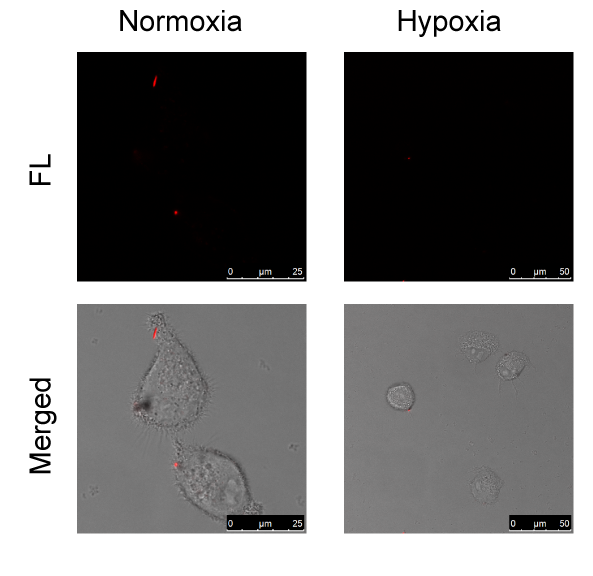


**Figure. S6** Imaging of HeLa cells under normoxia and hypoxia conditions**.** Note: The hydrophobic probe Azo-QM-PN cannot penetrate the cell membrane efficiently after incubated with cells for 6 h.

1. Reconstructed 3D tumor spheroid


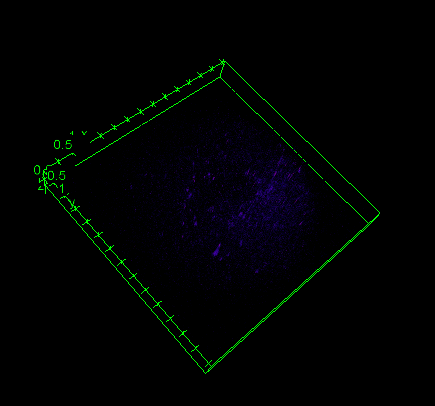


**Figure. S7** Reconstructed 3D tumor spheroid after treatment with Azo-QM-PN NPs.


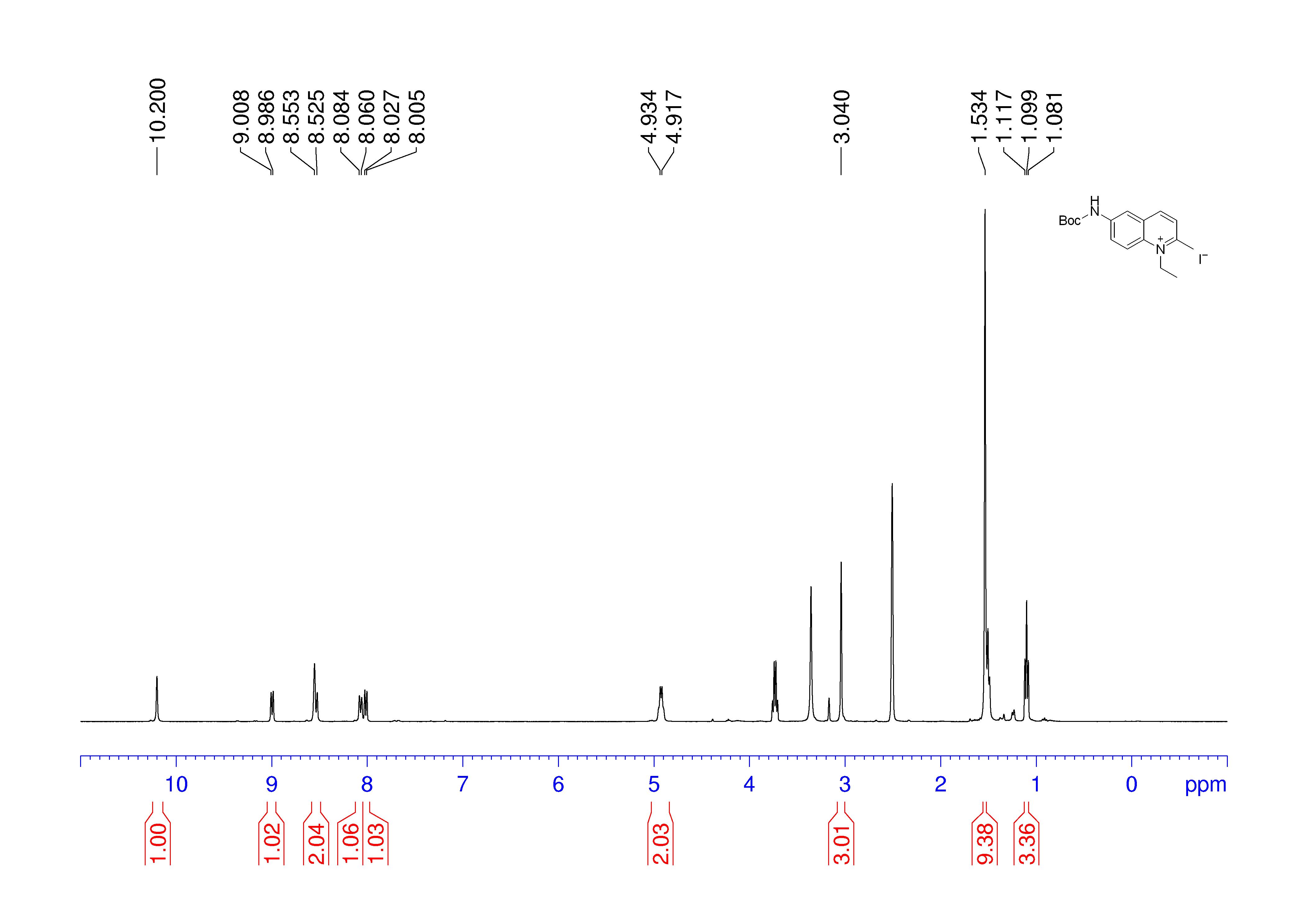


**Figure. S8** ^1^H NMR spectrum of Boc-QLI in DMSO-*d*_6_


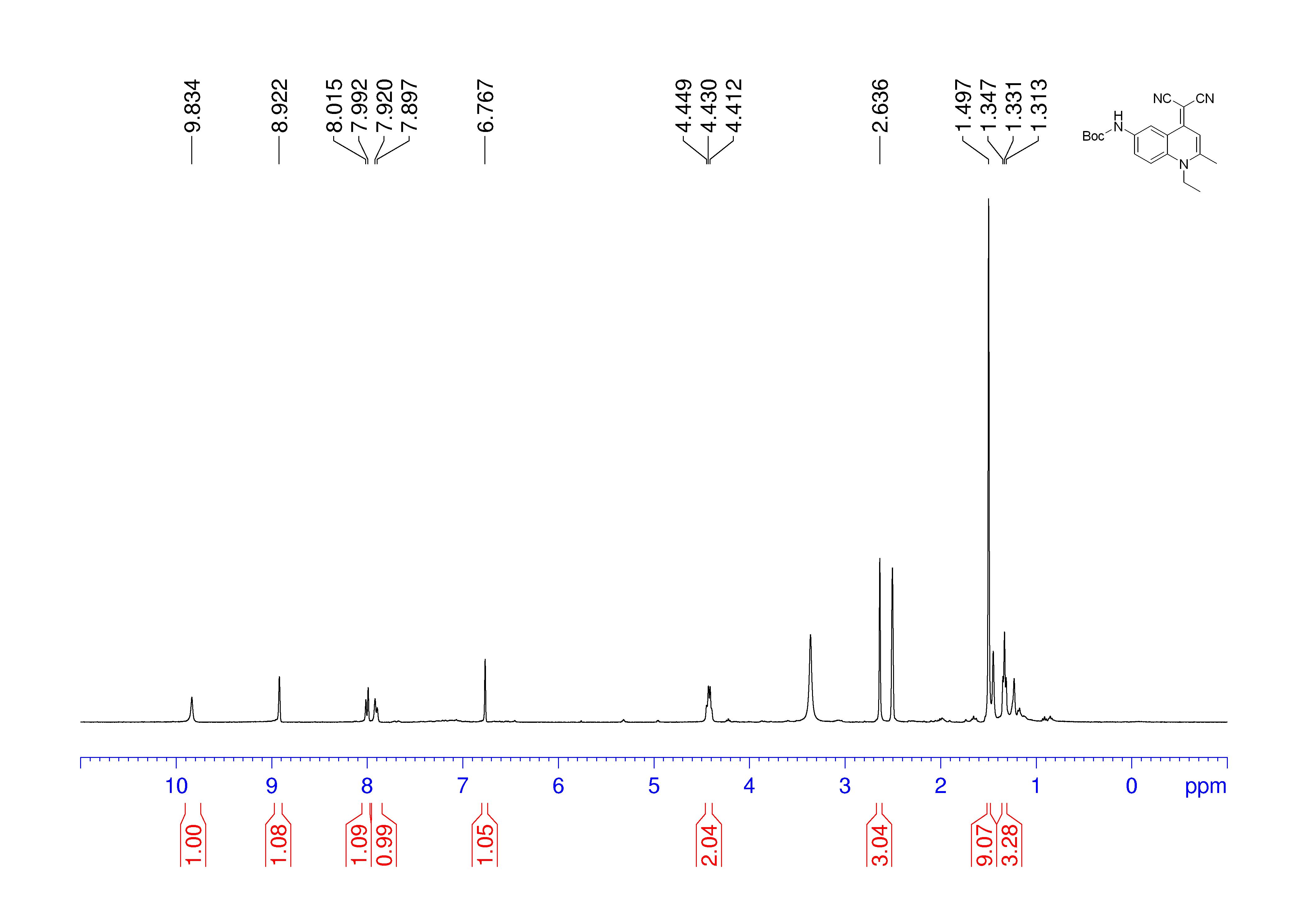


**Figure. S9** ^1^H NMR spectrum of Boc-QM in DMSO-*d*_6_


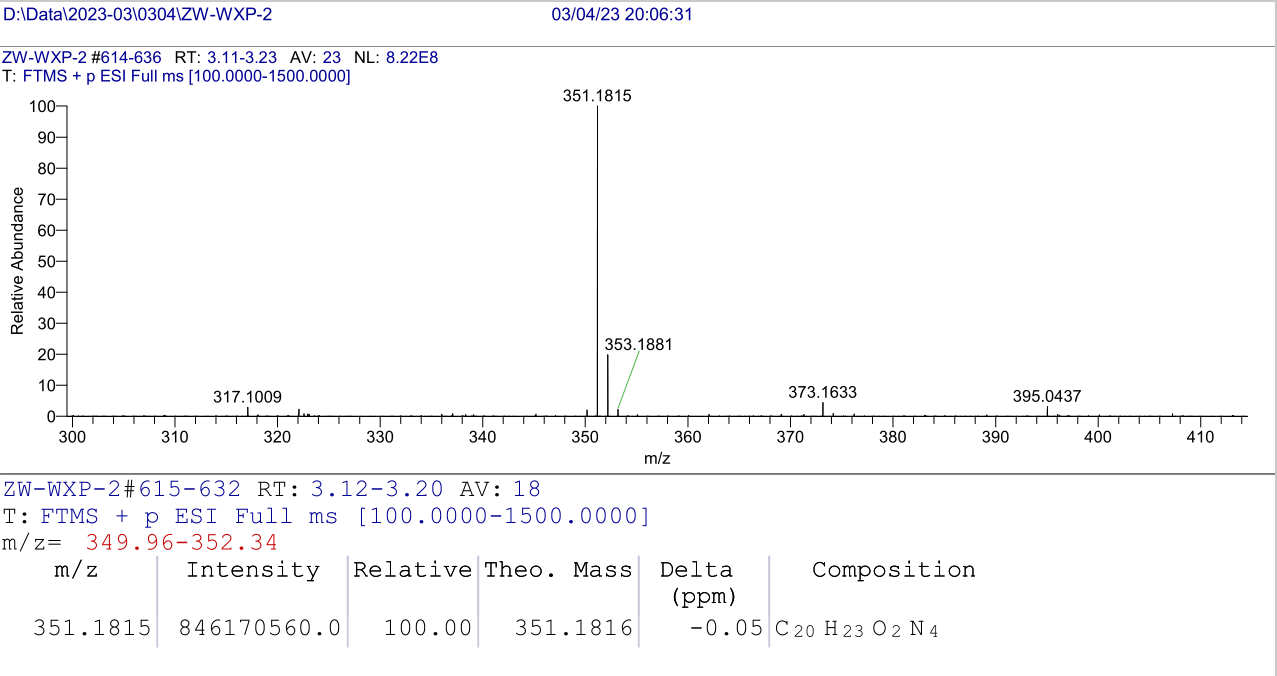


**Figure. S10** HRMS spectrum of Boc-QM


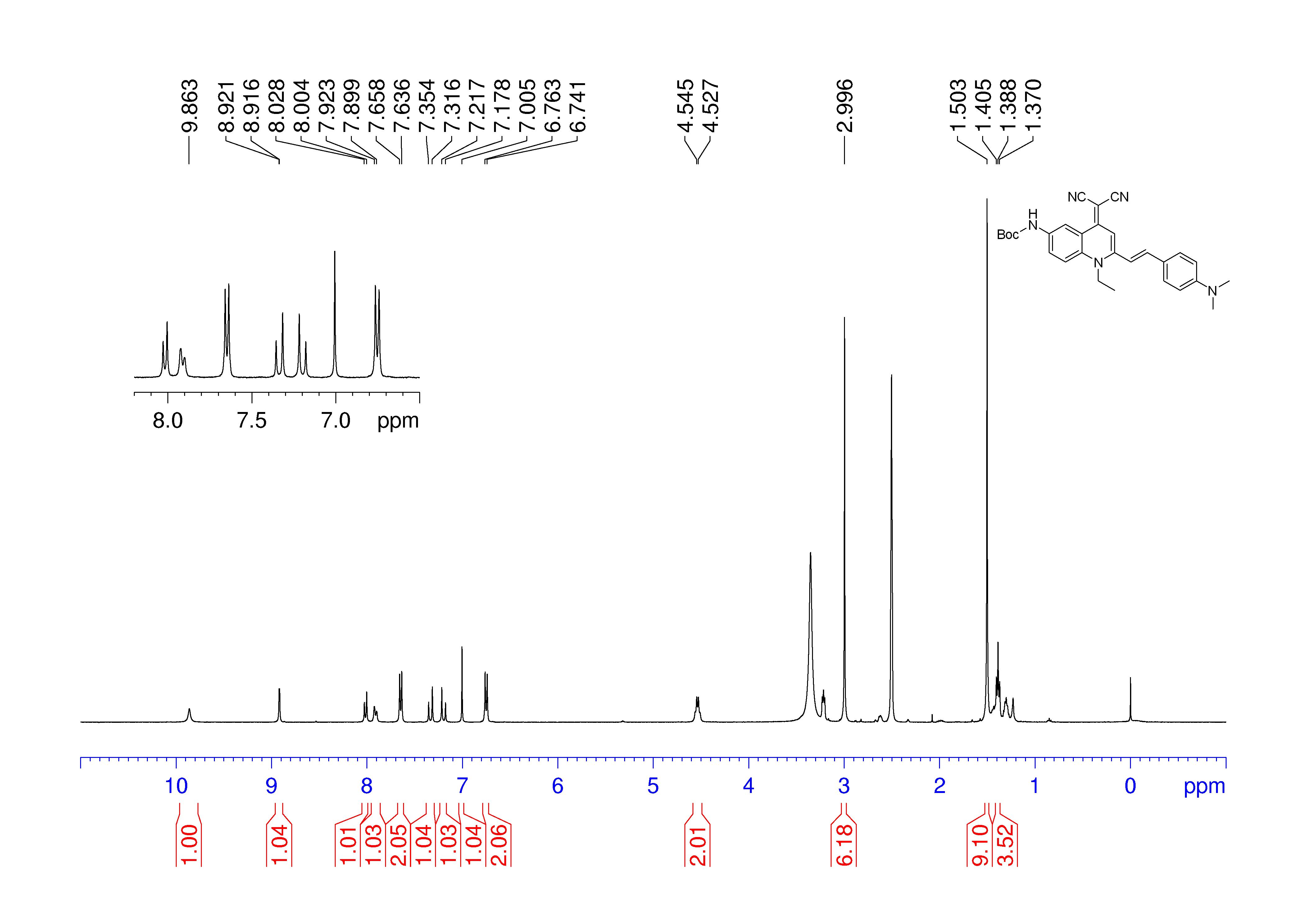


**Figure. S11** ^1^H NMR spectrum of Boc-QM-PN in DMSO-*d*_6_


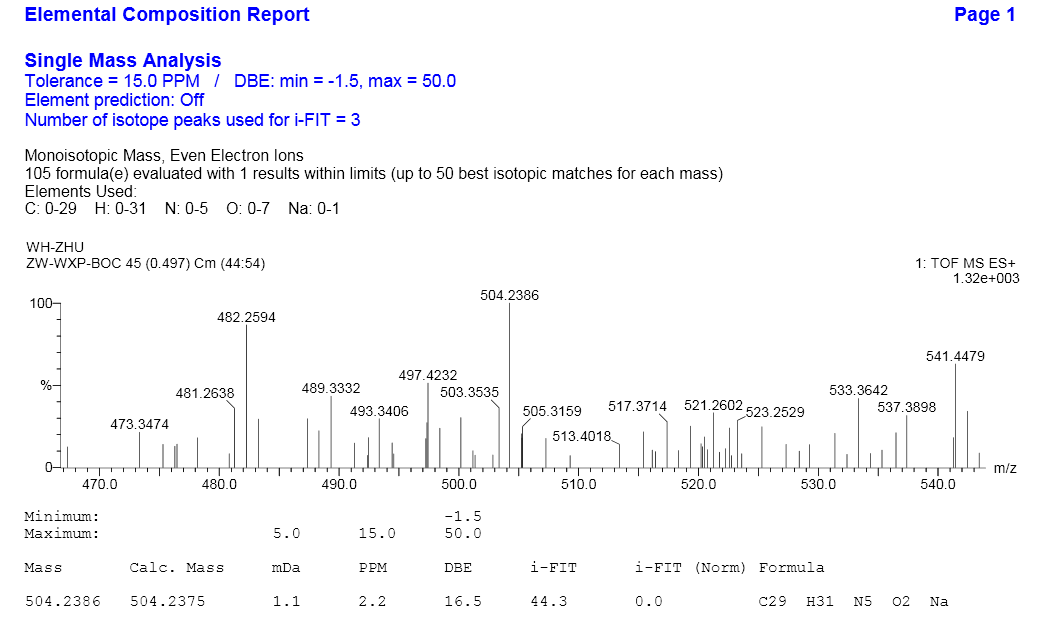


**Figure. S12** HRMS spectrum of Boc-QM-PN


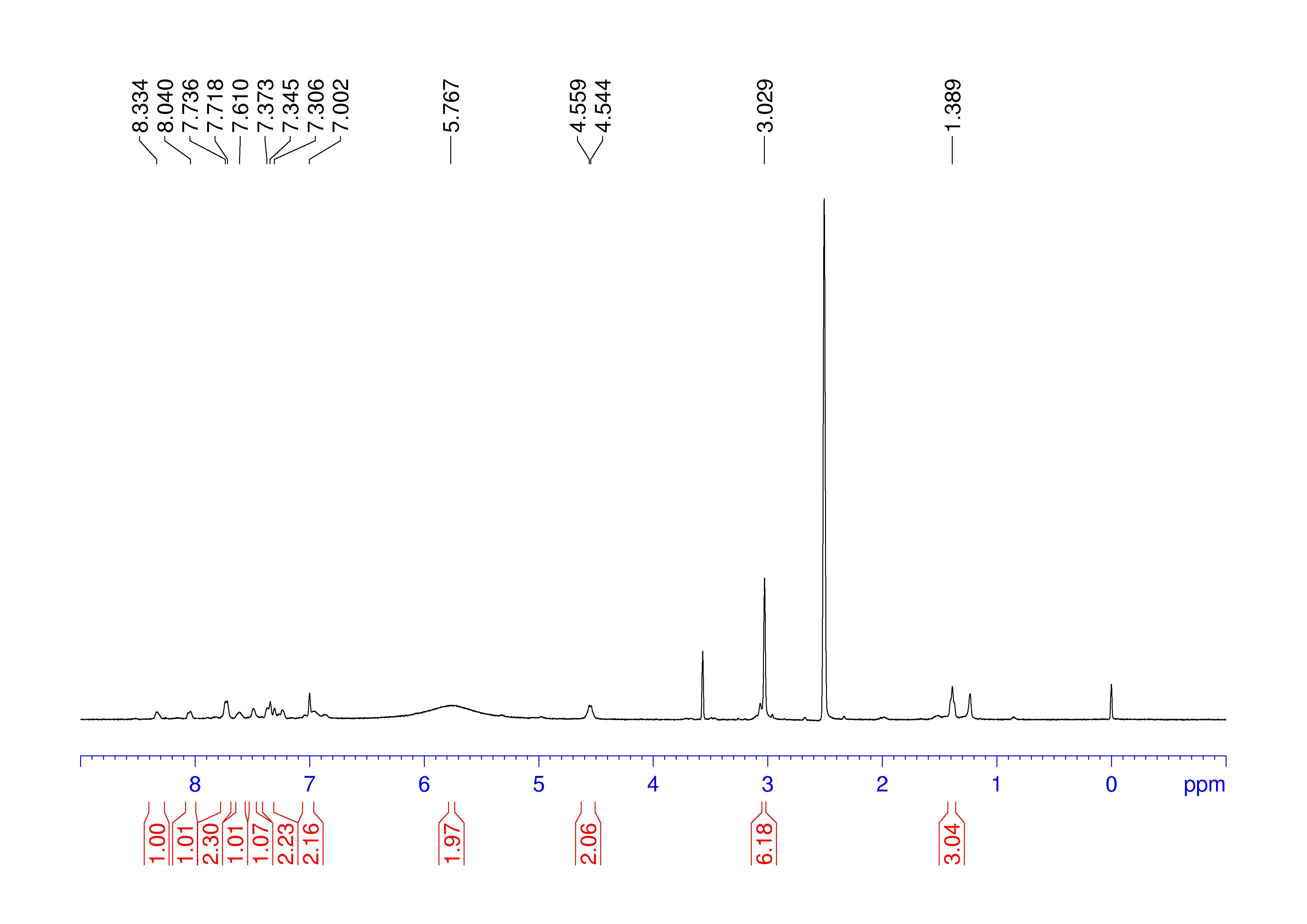


**Figure. S13** ^1^H NMR spectrum of NH_2_-QM-PN in DMSO-*d*_6_


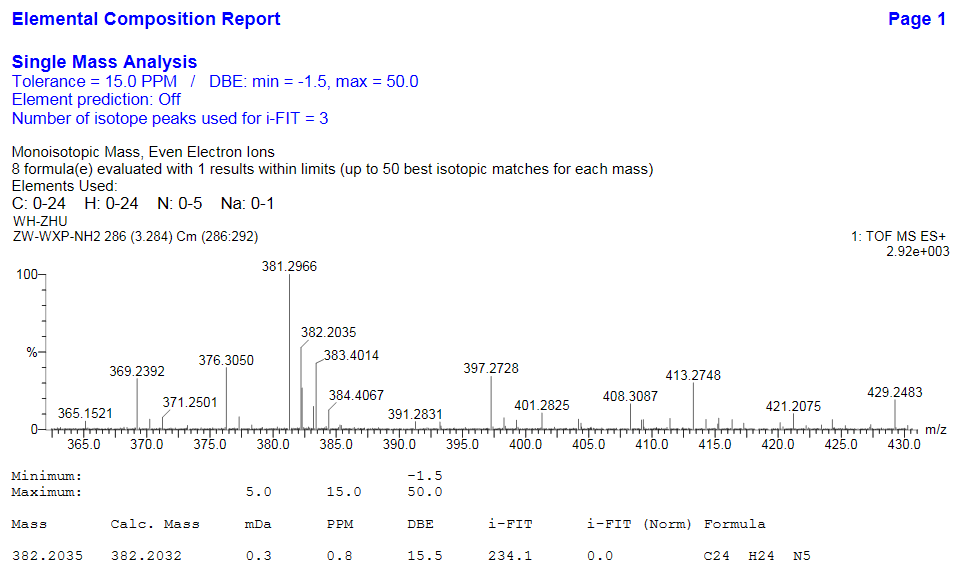


**Figure. S14** HRMS spectrum of NH_2_-QM-PN


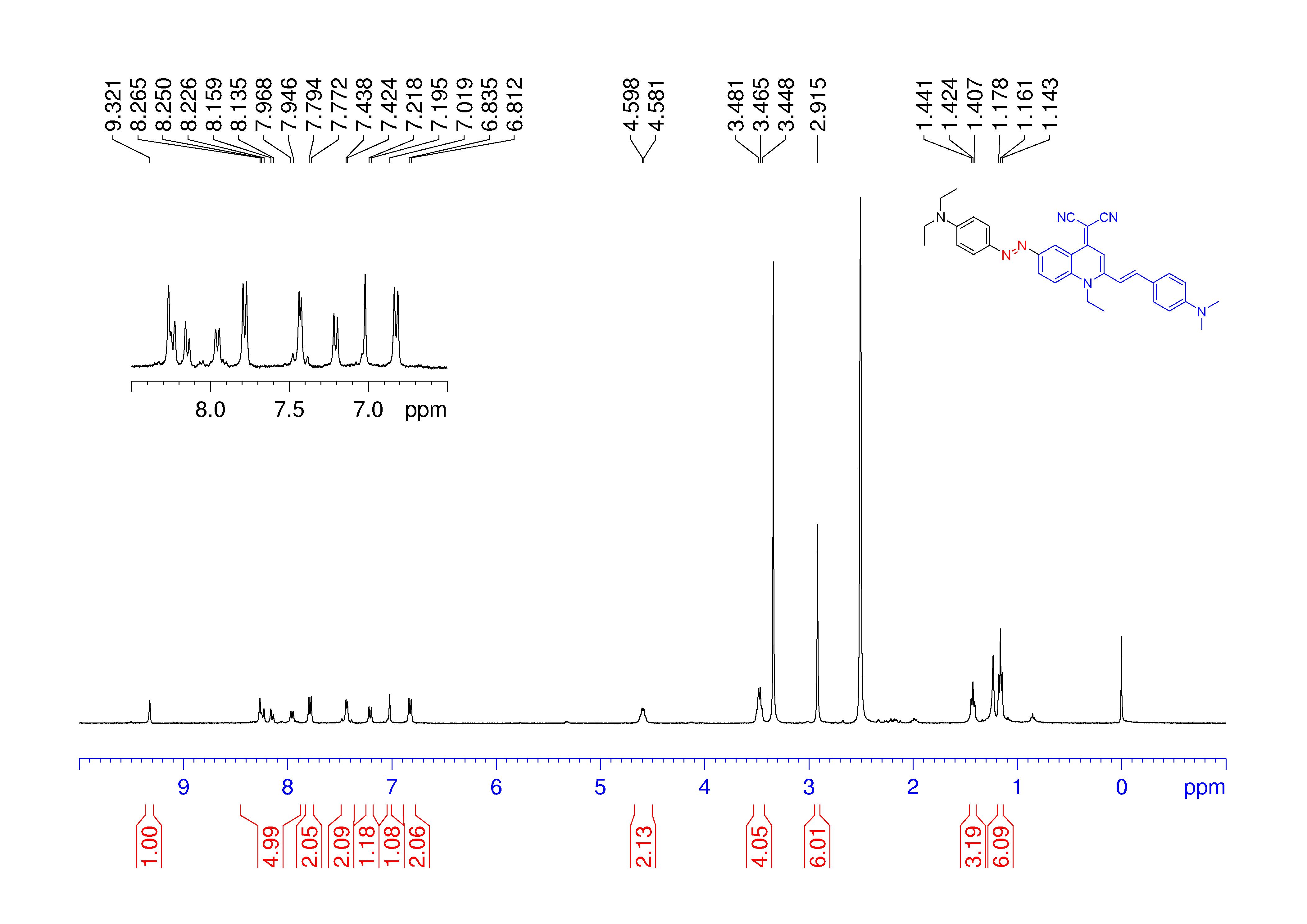


**Figure. S15** ^1^H NMR spectrum of Azo-QM-PN in DMSO-*d*_6_


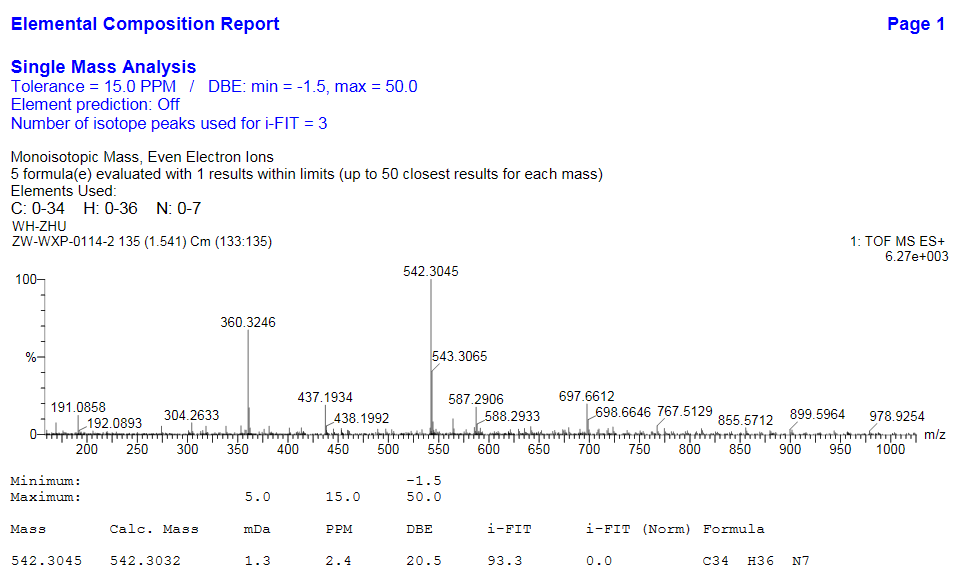


**Figure. S16** HRMS spectrum of Azo-QM-PN
